# Metabolomic and transcriptomic analyses of the flavonoid biosynthetic pathway in blueberry (*Vaccinium* spp.)

**DOI:** 10.3389/fpls.2023.1082245

**Published:** 2023-04-20

**Authors:** Yinping Li, Haifei Li, Shiyao Wang, Jing Li, Syed Asim Shah Bacha, Guofeng Xu, Jing Li

**Affiliations:** ^1^ Laboratory of Quality and Safety Risk Assessment for Fruit, Research Institute of Pomology, Chinese Academy of Agricultural Sciences, Xingcheng, Liaoning, China; ^2^ Department of Applied Biosciences, Toyo University, Ora-gun, Japan

**Keywords:** blueberry, PacBio sequencing, flavonoid, transcriptome, metabolite

## Abstract

As a highly economic small fruit crop, blueberry is enjoyed by most people in terms of color, taste, and rich nutrition. To better understand its coloring mechanism on the process of ripening, an integrative analysis of the metabolome and transcriptome profiles was performed in three blueberry varieties at three developmental stages. In this study, 41 flavonoid metabolites closely related to the coloring in blueberry samples were analyzed. It turned out that the most differential metabolites in the ripening processes were delphinidin-3-*O*-arabinoside (dpara), peonidin-3-*O*-glucoside (pnglu), and delphinidin-3-*O*-galactoside (dpgal), while the most differential metabolites among different varieties were flavonols. Furthermore, to obtain more accurate and comprehensive transcripts of blueberry during the developmental stages, PacBio and Illumina sequencing technology were combined to obtain the transcriptome of the blueberry variety Misty, for the very first time. Finally, by applying the gene coexpression network analysis, the darkviolet and bisque4 modules related to flavonoid synthesis were determined, and the key genes related to two flavonoid 3′, 5′-hydroxylase (*F3′5′H*) genes in the darkviolet module and one *bHLH* transcription factor in the bisque4 module were predicted. It is believed that our findings could provide valuable information for the future study on the molecular mechanism of flavonoid metabolites and flavonoid synthesis pathways in blueberries.

## Introduction

Blueberry, an economically important small fruit, belongs to the section *Cyanococcus* of the genus *Vaccinium*, family *Ericaceae* (Cho et al., 2017; Hou et al., 2017; Li X et al., 2020; Guo et al., 2021). The year of 1906 saw its beginning of commercial cultivation; since then, blueberry has been widely planted around the world (Brevis et al., 2008; Bian et al., 2014). It features a unique flavor and rich nutrition (Yuan et al., 2020; Zang et al., 2021). It contains sugar, organic acid, various amino acids, vitamins, unsaturated fatty acids, and minerals. More importantly, it is an excellent source of active substances such as anthocyanin, ascorbic acid, ellagic acid, and pterostilbene, which can be useful when it comes to improving eyesight, softening blood vessels, fighting bacteria and inflammation, inhibiting the growth of cancer cells, and resisting oxidation (Hou et al., 2017; Morita et al., 2017; Munagala et al., 2017; Wang et al., 2017; Lin et al., 2018). The antioxidant capacity of blueberry is mainly attributed to flavonoids, including flavonols, anthocyanins, and proanthocyanidins (Lin et al., 2018). Anthocyanin, one of the key quality factors in blueberries, can increase its commercial value. The common anthocyanins in blueberries are delphinidin, cyanidin, peonidin, petunidin, and malvidin (Chai et al., 2021). Anthocyanin mainly exists in the form of glycoside, and the glycosides include arabinose, glucose, galactose, and xylose (Hosseinian and Beta, 2007; Timmers et al., 2017; Zhou et al., 2018).

Flavonoids are synthesized starting from the phenylalanine pathway in the plant cytoplasm (Zifkin et al., 2012; Hassani et al., 2020). The molecular synthesis mechanism of flavonoids has been revealed in depth, including the structural genes directly encoding key enzymes in each step of the flavonoid synthesis pathway (Cho et al., 2016; Zhao et al., 2019; Liu et al., 2020). As a substrate, phenylalanine is catalyzed by phenylalanine ammonia lyase (PAL), cinnamate 4-hydroxylase (C4H), 4-coumarate-CoA ligase (4CL), chalcone synthase (CHS), chalcone isomerase (CHI) enzymes to synthesize flavanones. Under the catalysis of flavanone 3-hydroxylase (F3H) and flavonoid 3 ‘- hydroxylase (F3’H), flavanone forms dihydroflavonol, which is the most important intermediate metabolite in the flavonoid synthesis pathway. Dihydroflavonol 4-reductase (DFR), leucoanthocyanidin dioxygenase (LDOX), anthocyanin synthase (ANS), and UDP-glucose flavonoid 3-*O*-glucosyltransferase (UFGT) are responsible for dihydroflavonols to anthocyanins. Flavonol synthase (FLS) directs the oxidation of dihydroflavonol to produce flavonol. In addition, leucoanthocyanidins and anthocyanidins produce proanthocyanidins under the catalysis of leucoanthocyanidin reductase (LAR) and anthocyanin reductase (ANR), respectively (Tohge et al., 2017; Karppinen et al., 2021). Flavonoid glycosides are thought to be synthesized in the cytoplasm and transported to the vacuole through transport mechanism (including an ABC transporter) (Liu et al., 2020).

The biosynthesis and inhibition of flavonoids are affected by many factors, such as light, temperature, pH, and phytohormones (Liu et al., 2018; Li G et al., 2019). Flavonoid biosynthesis genes are largely regulated at the transcriptional level. The MYB-bHLH-WD40 (MBW) regulatory complex, composed of the *R2R3-MYB* transcription factor, *bHLH* transcription factor, and WD40 protein, has been shown that it could synergistically activate multiple genes for anthocyanin production in all angiosperms to date (Zhao et al., 2019; Li Y et al., 2020; Sun et al., 2020). In this MBW complex, the *MYB* transcription factor is core to determining the intensity and the pattern of anthocyanin production in plants (Castillejo et al., 2020). Moreover, another type of *R2R3-MYB* transcription factor can act alone with the structural genes of flavonoids (Naik et al., 2022). The negative regulation of flavonoids involves the participation of a variety of inhibitors, namely, *R2R3-MYB*, *R3-MYB*, *NAC*, miRNA, *HD-ZIP*, and so forth. They inhibit the syntheses of flavonoids by inhibiting the expression of the MBW complex or destroying the stability of the MBW complex (LaFountain and Yuan, 2021; Naik et al., 2022).

The genome of blueberry is relatively large. Commercial blueberry varieties cover several species and interspecific hybrids, including tetraploid highbush blueberry, diploid and tetraploid lowbush blueberry (*V. myrtilloides* and *V. angustifolium*), hexaploid rabbiteye blueberry (*V*. *virgatum*), and hybrids thereof (Rowland et al., 2012; Ge et al., 2019). To further study the molecular synthesis and regulation of flavonoids, transcriptome sequencing is considered to be an effective method. So far, transcriptome sequencing has been used to study the genes of the flavonoid synthesis pathway in potatoes (Cho et al., 2016), grapes (He et al., 2010), mulberry (Zhao et al., 2019), and so forth. PacBio single-molecule long-reads sequencing technology (SMRT) breaks through the reading length restriction of next-generation high-throughput sequencing (NGS) and has been applied to effectively capture full-length sequences of genomes and transcripts (Hoang et al., 2017; Chao et al., 2018). Transcriptome and metabolomics are combined to study the regulatory network between genes and metabolites. In this study, the samples from three developmental stages of the tetraploid southern highbush blueberry Misty were sequenced by SMRT to obtain a more comprehensive and accurate full-length transcriptome sequence of blueberry fruit. Moreover, we used the Illumina sequencing technique to obtain the transcriptomes of Duke and Emerald at three developmental stages and analyzed all the transcriptome sequencing results of the three blueberry varieties to identify the important regulatory genes and possible pathways related to their ripening. Finally, we combined the metabolic data of flavonoids in these three blueberry cultivars to further screen the key genes and verify the potentially related genes, providing a molecular basis for the study of blueberry fruit color.

## Materials and methods

### Plant samples

**Figure 1:**
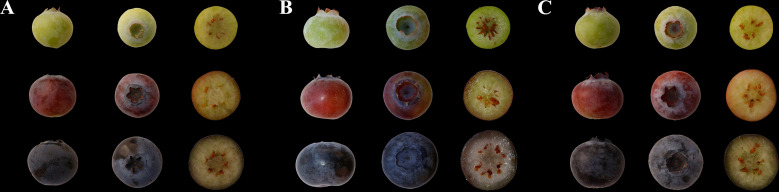
Green, pink, and blue stages of Misty, Duke, and Emerald. **(A)** Misty, **(B)** Duke, and **(C)** Emerald.

Three blueberry varieties Misty, Duke, and Emerald were collected in sheds from Niuyingzi village, Wanghai Township, Xingcheng City, Liaoning Province, China, in May 2018 (**Figure 1**). Three developmental stages of whole blueberry fruits, namely, green, pink, and blue, were sampled and immediately frozen in liquid N_2_ and stored at −80°C for flavonoid analysis and RNA extraction.

The RNAs of nine samples (three biological replicates per developmental stage) of Misty were mixed three replicates of each developmental stage with equal volume and sequenced on the PacBio RS II platform, while the RNAs of 18 samples of Duke and Emerald were subjected to 150 bp paired-end sequencing using the Illumina HiSeq X Ten platform.

### Chemical reagents

HPLC-grade acetonitrile and methanol were purchased from Thermo Fisher Scientific (Fair Lawn, NJ, USA). Formic acid of LC-MS grade was purchased from Anaqua Chemicals Supply (Shanghai, China). Flavonoids, including 13 flavonols and 28 anthocyanins, were purchased from Sigma–Aldrich Company Ltd. (Shanghai, China), ChromaDex Corp (Santa Anna, CA, USA), and PhytoLab company (Vestenbergsgreuth, Germany) (**Supplementary Tables S1**, **S2**).

### Measurement of flavonoid

The extraction of flavonoids was carried out in a way that described in a previous study with minor modifications (Appelhagen et al., 2011; Vrhovsek et al., 2012). The obtained filtrate was dissolved to 5 ml and filtered through a 0.22-μm organic phase filter membrane before UPLC–MS/MS analysis.

Metabolite profiling was conducted using an Acquity UPLC system. The system was interfaced to a Xevo triple-quadrupole mass spectrometer and was equipped with an electrospray ionization source operating in positive ionization modes (Waters Corp., Milford, MA, USA). Chromatographic separation was performed on a Waters ACQUITY UPLC^®^ HSS T3 column (2.1 mm × 150 mm, 1.8 μm) with a column temperature of 40°C. The mobile phase was composed of solvent A (methanol: acetonitrile, 7:3) and solvent B (5% formic acid solution) at a flow rate of 0.3 ml/min. The following gradient elution program was run: 0–1 min, 10% A; 1–16 min, 25% A; 16–18 min, 40% A; 18–19 min, 80% A; 19–20 min, 10% A. The injection volume was 2 μl. The MS/MS parameters were used: ion source temperature of 150°C, desolvation temperature of 500°C, desolvent gas flow of 800 liter/h, cone gas flow of 50 liter/h, collision gas (high-purity argon) flow, and 0.13 ml/min (**Supplementary Tables S1**, **S2**).

The acquisition was performed in multiple reaction monitoring (MRM) mode. The metabolite data were analyzed by principal component analysis (PCA) and the orthogonal partial least-squares-discriminant analysis (OPLS-DA) modules with SIMCA14.1 software. The predictive variable importance for the projection larger than 1 (VIPpred > 1) was considered to be the differential metabolite.

### RNA extraction, cDNA library construction, and sequencing

Total RNA was isolated with PureLink^®^ Plant RNA reagent (Life Technologies, Carlsbad, CA, USA) following the standard protocol. The extracted RNA was checked on 1% agarose gels for degradation and contamination and then measured using a Qubit^®^ RNA Assay Kit in a Qubit^®^2.0 Fluorometer (Life Technologies, Carlsbad, CA, USA) for its concentration. The RNA was qualified with a NanoPhotometer^®^ spectrophotometer (IMPLEN, CA, USA) and an Agilent Bioanalyzer 2100 system (Agilent Technologies, Santa Clara, CA, USA) before library construction.

A total amount of 5 μg of total RNA was used to synthesize cDNA using the SMARTer^®^ PCR cDNA Synthesis Kit (Takara Biotechnology, Dalian, China). Input DNA damage and end repatriation were treated with a SMRTbell Template Prep Kit (Pacific Biosciences, Menlo Park, CA, USA). Amplicons’ size selection was performed using the BluePippin™ Size Selection System (Sage Science, Beverly, MA, USA). SMRTbell template libraries were generated using the Pacific Biosciences DNA Template Prep Kit 2.0 before sequencing. The libraries were generated using the PacBio DNA/Polymerase Kit for sequencing primer annealing and polymerase binding. Subsequently, SMRT sequencing was carried out on a PacBio RSII instrument at the Biomarker Biotechnology Corporation (Beijing, China).

A total amount of 3-μg RNA per sample was used as an input material, and sequencing libraries were generated using the NEBNext^®^Ultra™ RNA Library Prep Kit for Illumina^®^ (NEB, Ipswich, MA, USA) following the manufacturer’s recommendation. Their ligated cDNA library was sequenced using the Illumina HiSeq™ 2000 system by Biomarker Biotechnology Corporation (Beijing, China).

### PacBio data analysis

PacBio raw reads were filtered out of low-quality reads and adaptor sequences using SMRT analysis v2.3.0 software. The reads of insert (ROI) sequences were extracted from original sequences, requiring a minimum read quality of 0.75 and a minimum of one full pass. Full-length (FL) reads and non-full-length reads (NFL reads) were identified using the RS module in SMRT Analysis software by detecting the existence of 3′ and 5′ primers, as well as the polyA tail in ROLs. FL reads contained all three elements, and full-length non-chimeric (FLNC) reads were regarded as no additional copies of adapter sequence within FL reads. Consensus isoforms were obtained by using the ICE (Iterative Clustering for Error Correction) algorithm to cluster FL reads into a cluster. Then, combined with NFL reads, the consistent sequences in each cluster were polished by the quiver program and, finally, high-quality isoforms with more than 99% postcorrection accuracy were obtained. Additionally, read corrections were carried out by using Illumina RNA-Seq reads of the same experiment with Proovread v2.12 (Rodriguez-Mateos et al., 2012), resulting in the corrected isoforms.

The transcripts were annotated by MISA software for SSR analysis and TransDecoder software for predicting the potential coding sequences and corresponding amino acid sequences of transcripts. The lncRNA identification from isoform sequencing was calculated by the four common used approaches, including CPC analysis (Li et al., 2014; Cho et al., 2017), CNCI analysis (Altschul et al., 1997), Pfam protein domain analysis, and CPAT analysis (Deng et al., 2006). Transcription factors in blueberries were analyzed with iTAK software (Zheng et al., 2016). Using Blast v2.2.26 software (Altschul et al., 1997), the obtained nonredundant transcripts were compared with the NR (Deng et al., 2006), SwissProt (Apweiler, 2004), GO (Ashburner et al., 2000), COG (Tatusov et al., 2000), KOG (Koonin et al., 2004), eggNOG (Huerta-Cepas et al., 2016), and Kyoto Encyclopedia of Genes and Genomes (KEGG) databases (Huerta-Cepas et al., 2016) to obtain annotation information for the transcripts.

### Illumina data analysis

RNA-seq and *de novo* transcriptome assembly in blueberries were carried out as described by Huerta-Cepas (Wu et al., 2016). The expression analysis was performed with RESM software based on the FPKM (fragments per kilobases per million fragments) values (Li and Dewey, 2011). The differentially expression analyses between samples from different developmental stages were performed by the DESeq R package (Leng et al., 2013) based on a mold of the negative binomial distribution. Genes with FDR < 0.01 and fold change ≥ 2 were identified as differentially expressed genes (DEGs). GO enrichment analysis of DEGs was implemented by the topGO R packages based on the Kolmogorov–Smirnov test. KEGG pathways of DEGs were constructed using KOBAS software (Mao et al., 2005).

### WGCNA, KEGG, and candidate hub genes analysis

For coexpression network analysis, we conducted weighted gene coexpression network analysis (WGCNA) with the default settings in BMKCloud (http://www.biocloud.net). To obtain the genes related to flavonoid synthesis, all DEGs and metabolomics data detected in the three developmental stages of blueberries were subjected to WGCNA to construct trait-related modules. Additionally, the meaningful modules were annotated with KEGG pathway enrichment analysis by ggplot on the BMKCloud platform. The coexpression network was visualized using Cytoscape software (version 3.7.1). The cytoHubba app was used to find the top 20 hub genes based on the maximal clique centrality (MCC) algorithm.

## Results

### Metabolic profiling of blueberry

To understand the color formation of blueberry during ripening, UPLC–MS/MS was used to detect 13 flavonols and 28 anthocyanins in the three blueberry cultivars (**Figure 1**). All metabolites were analyzed by PCA and OPLS-DA modules.

**Figure 2:**
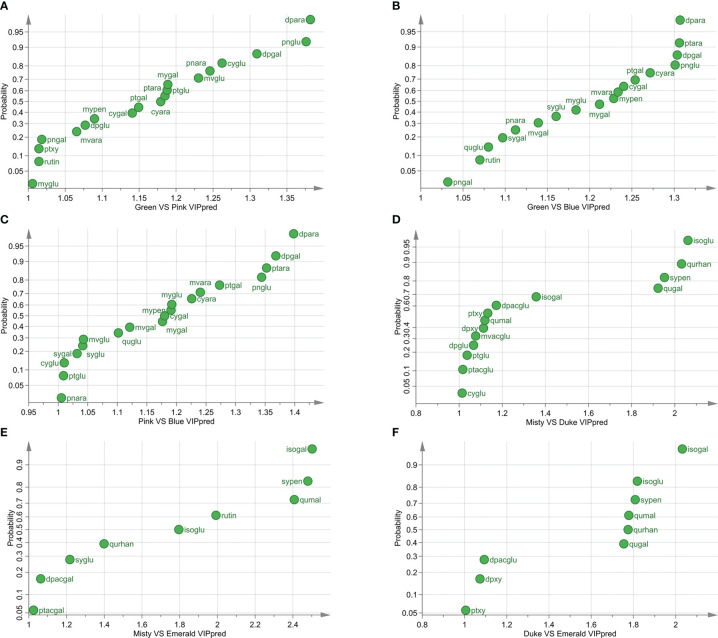
Different metabolites of three varieties at different developmental stages. **(A)** Differential metabolites of three blueberry varieties at the green and pink stages (VIP > 1). **(B)** Differential metabolites of three blueberry varieties at the green and blue stages (VIP > 1). **(C)** Differential metabolites of three blueberry varieties at the pink and blue stages (VIP > 1). **(D)** Differential metabolites of Misty and Duke in three developmental stages (VIP > 1). **(E)** Differential metabolites of Misty and Emerald in three developmental stages (VIP > 1). **(F)** Differential metabolites of Duke and Emerald in three developmental stages (VIP > 1). VIPpred > 1, predictive variable importance for the projection larger than 1.

First, the metabolite differences at different developmental stages were studied. The pairwise comparison of the three varieties at different stages showed that the difference of metabolites between the green and blue fruit stages was the most significant (**Figures 2A–C**; **Supplementary Tables S1**, **S2**). In the green and pink fruit stages, 19 differential metabolites (VIPpred > 1) in the three varieties were found (**Figure 2A**). In the green and blue fruit stages, 18 differential metabolites among the three varieties were discovered (**Figure 2B**). In the pink and blue fruit stages, there were 19 differential metabolites among the three varieties (**Figure 2C**). Among the three blueberry varieties, 12 identical metabolites were found in the three developmental stages of fruit ripening, and the highest VIPpred values were delphinidin-3-*O*-arabinoside (dpara), peonidin-3-*O*-glucoside (pnglu), and delphinidin-3-*O*-galactoside (dpgal).

Then, we compared the metabolite differences among blueberry varieties during fruit ripening and found that the main differential metabolite of blueberry fruits was flavonols (**Figures 2D–F**; **Supplementary Tables S1**, **S2**). The results of pairwise comparisons among the three blueberry varieties during the fruit ripening process showed that there were 14 differential metabolites between Misty and Duke, nine differential metabolites between Misty and Emerald, and nine differential metabolites between Duke and Emerald (**Figures 2D–F**). Additionally, five identical metabolites among the three blueberry cultivars were isorhamnetin-3-*O*-galactoside (isogal), syringetin-3-*O*-pentoside (sypen), quercetin-3-*O*-malonylgluctoside (qumal), isorhamnetin-3-*O*-gluctoside (isoglu), and quercetin-3-*O*-rhanosyl-galacoside (qurhan).

### PacBio sequencing and functional annotation

To obtain a comprehensive catalog of the southern highbush blueberry transcriptome, PacBio technology was employed to sequence the transcriptomes of the three developmental stages of Misty fruits. A pooled sample representing high-quality RNAs from different developmental stages of Misty fruits was sequenced, and three size-fractionated libraries (1–2, 2–3, and 3–6 kb) were constructed to avoid loading bias. Size-fractionated libraries (1–2, 2–3, and 3-6 kb) were constructed and sequenced using the PacBio RS II platform with nine cells for each mixed sample (**Supplementary Figure S1A**), yielding 15.24 Gb data. A total of 675,483 ROIs were generated, including 232,368 in 1–2 kb libraries, 264,200 in 2–3 kb libraries, and 178,915 in 3–6 kb libraries. As expected, the mean length of the ROIs was consistent with each size-selected library, with an average length of 3435.33 bp. Of these ROIs, 285,909 FL reads were identified based on the presence of 5′ primers, 3′ primers, and poly (A) tails. Additionally, 284,329 FLNC reads were identified with 0.55% artificial connectors (**Supplementary Figure S1B**). In total, 158,832 consensus isoforms (27,102 polished high-quality isoforms and 131,718 polished low-quality isoforms) were obtained from isoform-level clustering and quiver polishing, which were further corrected with Illumina RNA-seq data (**Supplementary Figure S1C**).

After removing the redundant sequences for all SMRT subreads using Cogent software, 57,220 nonredundant reads were produced. Using transcoder software to predict the coding region, the results showed that a total of 55,143 ORFs were obtained, of which 34,313 were complete ORFs. After the sequence-structure analysis of nonredundant reads, 43,868 SSRs and 34,313 complete CDS regions were predicted (**Supplementary Figures S2A**, **S2B**). After filtering through the four approaches (CPC, CNCI, PLEK, and Pfam), we finally obtained 1,295 long noncoding RNAs (lncRNAs) (**Supplementary Figure S2C**). Then, the LncTar target gene prediction tool was used to predict the target genes of lncRNAs, and a total of 1,293 novel lncRNAs were targeted. Moreover, a total of 5,492 transcription factors were identified from the Iso-Seq reads, including 2,223 transcription factors, 883 transcript regulators, and 2,454 protein kinases, which belong to 20 families of transcription factors (**Supplementary Figure S2D**). To analyze the functions of the transcripts, we annotated 54,585 nonredundant transcripts using the data from seven nucleotide and protein databases (**Supplementary Figures S3**).

### Illumina sequencing and identification of the differentially expressed genes

In this study, to understand the molecular basis of the metabolic differences detected in the three different developmental stages, transcriptome sequencing of 27 samples was carried out using IIumina sequencing technique. After removing the adaptor and low-quality sequence, a total of 178.03 G of clean data was generated, and the Q30 values of each sample were not less than 92.53%. In this project, nonredundant transcripts obtained by SMRT sequencing were used as references for sequence alignment and subsequent analysis. Clean reads were sequence-aligned with transcripts by using STAR to obtain location information on the transcripts. Using RSEM software (Li and Dewey, 2011), FPKM was used as the index to measure the expression level of transcripts or genes through the location information of mapped reads on the third-generation transcripts. Taking Pearson correlation coefficient (R) as the evaluation index of biological repeat correlation, the correlation coefficients of the biological replicates at the same developmental stage of the same blueberry cultivar were higher than 0.95, indicating that the reproducibility of the biological replicates was satisfied. These results showed that the quantity and quality of the sequencing data were high enough to guarantee further analysis.

Pairwise comparisons of each sample detected 205,930 DEGs by DESeq software (**Supplementary Figure S4**). First, the DEGs of the same blueberry variety at different developmental stages were analyzed. In the comparison of MGvsMP, MGvsML, and MPvsML, 2,539, 4,125, and 633 significant DEGs were identified. In the comparison of DGvsDP, DGvsDL, and DPvsDL, 5,546, 7,619, and 677 DEGs were found out. In the comparison of EGvsEP, EGvsEL, EPvsEL, 4,389, 6,342, and 887 DEGs were discovered. We also identified DEGs among different varieties at the same developmental stage. In the comparison of DGvsEG, DGvsMG, and EGvsMG, 20,011, 18,605, and 18,774 DEGs were obtained. In the comparison of DPvsEP, DPvsMP, and EPvsMP, 20,450, 19,086, and 18,146 DEGs were obtained. In the comparison of DBvsEB, DBvsMB, and EBvsMB, 20,795, 19,205, and 18,101 DEGs were obtained.

### Analyses of coexpression networks revealed flavonoid-related DEGs

**Figure 3:**
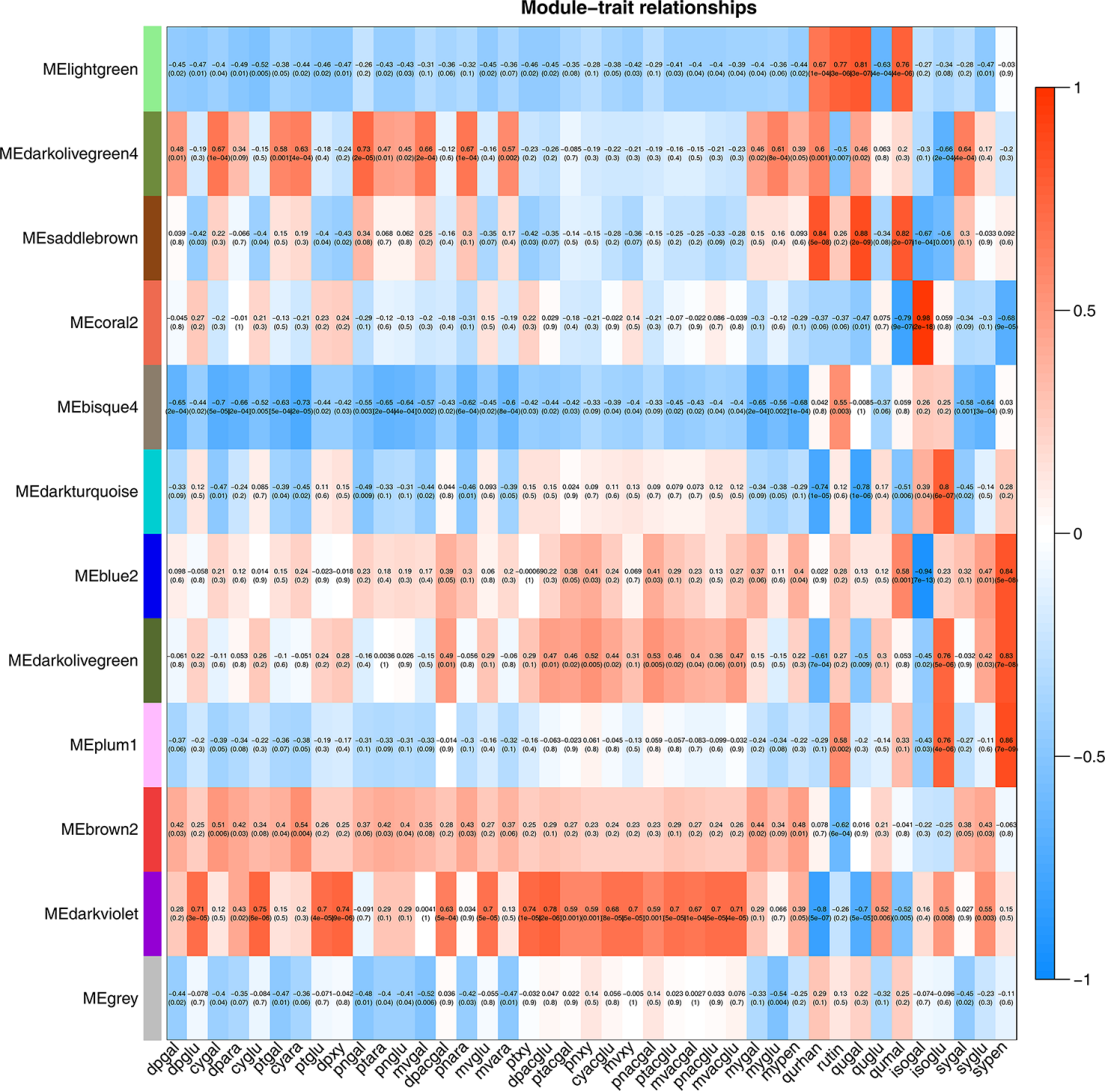
Weighted gene coexpression network analysis (WGCNA) of blueberry flavonoids. Module-flavonoid association. Each row corresponds to a module. Each column represents a specific flavonoid.

**Figure 4:**
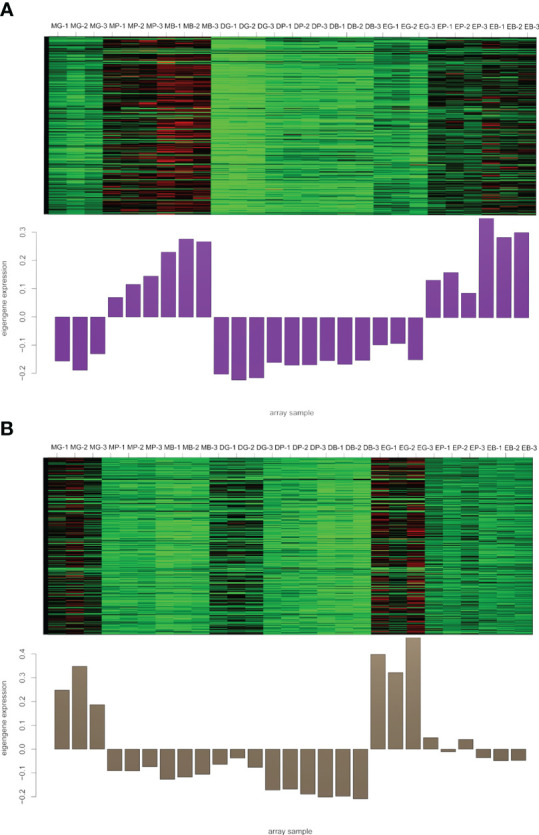
Heatmap of genes and eigengene expression in the darkviolet and bisque4 modules. **(A)** Heatmap of genes and eigengene expression in the darkviolet module. **(B)** Heatmap of genes and eigengene expression in the bisque4 module. DG, Duke green stage; DP, Duke pink stage; DB, Duke blue stage; EG, Emerald green stage; EP, Emerald pink stage; EB, Emerald blue stage; MB, Misty green stage; MP, Misty pink stage; MB, Misty blue stage.

To identify flavonoid biosynthesis-related transcripts, we performed WGCNA on all DEGs and identified 12 modules (**Figure 3**; **Supplementary Figure S5**). In the module-trait relationships, the modules showing the highest correlations with the synthesis of most flavonoid metabolites were selected, namely, darkviolet and bisque4 modules. Darkviolet was positively correlated with 36 metabolites, and bisque4 was negatively correlated with 34 metabolites. To identify gene expression patterns in the two modules, their expression patterns were shown in the heatmap (**Figure 4**). The heatmap and expression of key genes indicated that the two modules were divided into two groups. The expression patterns of Misty and Emerald’s three developmental stages were similar in these two modules, and different from the expression patterns of Duke’s three periods in these two modules, which implies a difference in the bioactive product contents between the southern and northern highbush blueberry varieties.

**Figure 5:**
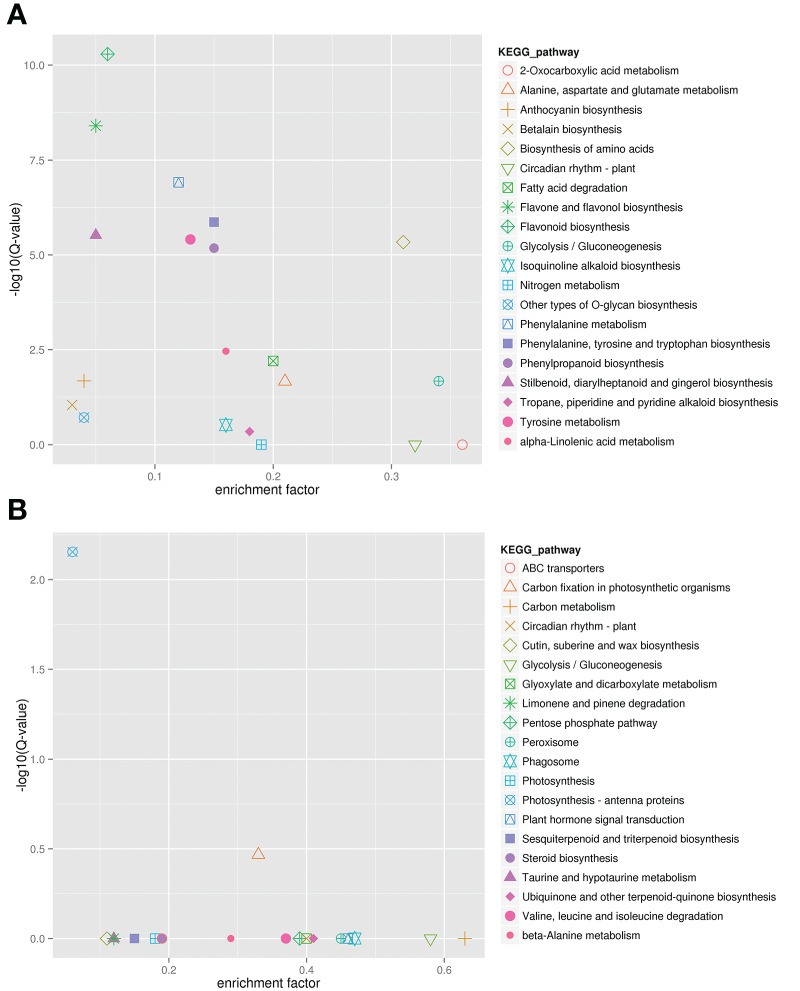
Kyoto Encyclopedia of Genes and Genomes (KEGG) annotation of darkviolet and bisque4 modules. **(A)** KEGG enrichment of darkviolet module. **(B)** KEGG enrichment of bisque4 module.

KEGG analysis was carried out to further determine the gene function of these two modules (**Figure 5**). The results showed that genes in the darkviolet and bisque4 modules were mainly related to the synthesis and metabolism of primary and secondary metabolites. From **Figure 5A**, genes in the darkviolet module were mainly enriched in phenylpropanoid, flavone, flavonol, and anthocyanin biosynthesis pathways. The abundant flavonoid synthesis structural genes in the darkviolet module further proved that the coexpression genes in this module were involved in the accumulation of flavonoids in blueberry fruits. From **Figure 5B**, genes in the bisque4 module were significantly enriched for photosynthesis-antenna proteins and carbon fixation in photosynthetic organization pathways.

### Structural genes involved in flavonoid synthesis

**Figure 6:**
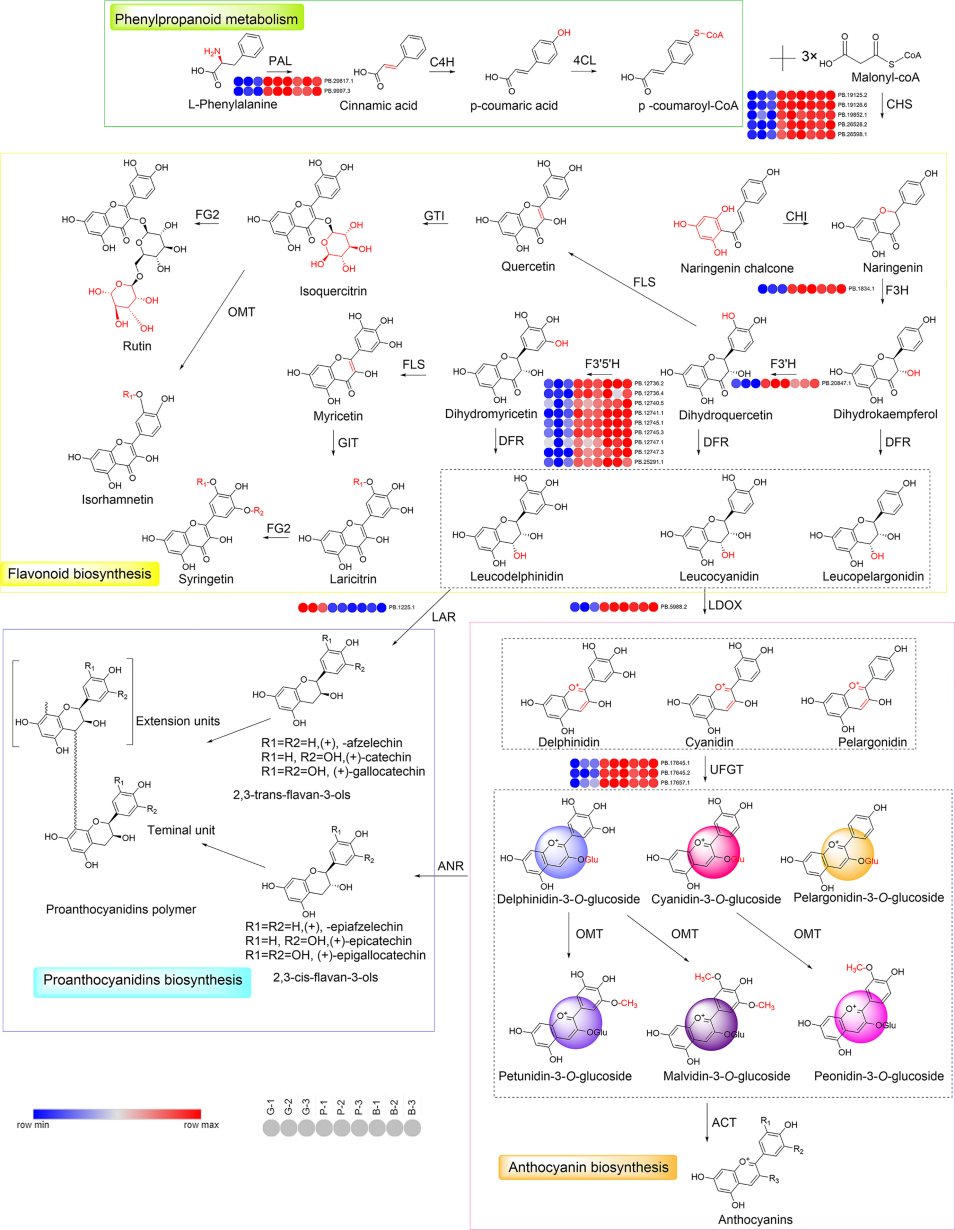
Reconstruction of the phenylpropanoid-flavonoid-anthocyanin biosynthesis pathway in Misty blueberry. The typical colors of anthocyanidins are also shown. *PAL*, phenylalanine ammonia lyase; *C4H*, cinnamate 4-hydroxylase; *4CL*, 4-coumarate-CoA ligase; *CHS*, chalcone synthase; *CHI*, chalcone isomerase; *F3H*, flavanone 3-hydroxylase; *F3*′*H*, flavonoid 3′-hydroxylase; *F3*′*5*′*H*, flavonoid 3′, 5′-hydroxylase; *FLS*, flavonol synthase; *GTI*, Anthocyanidin 5, 3-*O*-glucosyltransferase; *FG2*, flavonol-3-*O*-glucoside L-rhamnosyltransferase; *DFR*, dihydroflavonol 4-reductase; *OMT*, o-methyltransferase; *LDOX*, Leucoanthocyanidin Dioxygenase; *UFGT*, UDP-glucose flavonoid 3-*O*-glucosyltransferase; *LAR*, leucoanthocyanidin reductase; *ANS*, Anthocyanidin synthase; *ACT*, anthocyanin acyltransferase. G, green stage; P, pink stage; B, blue stage.

As shown in **Figures 4**, **6**, the structural and regulatory genes play an important role in flavonoid synthesis process. Structural genes are directly involved in the flavonoid pathway through the synthesis of specific enzymes (**Figure 6**). The structural genes in the two modules were analyzed respectively (**Figure S6**; **Supplementary Tables S3**, **S4**). In the darkviolet module, eight structural genes in the flavonoid pathway were revealed, namely, *PAL*, *C4H*, *CHS*, *F3H*, *F3*′*H*, *F3*′*5* ′*H*, *LDOX*, and *UFGT*, while in the bidsque4 module, only one flavonoid synthesis structural gene was disclosed, namely, *LAR*.

### Transcription factors involved in the synthesis of flavonoids

Transcription factors are involved in regulating various pathways of plant growth and development, and can also regulate the expression of specific genes in the flavonoid synthesis pathway (Li Y et al., 2020; Karppinen et al., 2021). At present, the reported transcription factors include *MYB*, *bHLH*, *bZIP*, *AP2/ERF*, *NAC*, and zinc finger (LaFountain and Yuan, 2021). In this study, the transcription factors related to flavonoid synthesis in darkviolet module include 1 *MYB*, 2 *MYB*-related, 1 *bZIP*, 4 *AP2/ERF-ERF*, 3 *NAC*, and 3 zinc fingers (**Supplementary Figure S6A**; **Tables S3**, **S4**). Through phylogenetic analysis, it is found that the *MYB* transcription factor named *VcMYBM1* belongs to the *R2R3-MYB* SG6 subfamily, which mainly regulates the synthesis of anthocyanins (**Supplementary Figure S7**; **Table S5**). More than 10 repressors to flavonoid synthesis were found, mainly the inhibitors of corresponding environmental signals or hormones, which directly or indirectly regulate MBW complex and structural genes in the flavonoid synthesis pathway through protein-protein interaction or post-transcriptional gene silencing (Xiao et al., 2022). In bisque4 module, there are 4 *bHLH*, 1 *bZIP*, 2 *DBB*, 1 *B3*, 1 *AUX/IAA*, 7 *HB-HD-ZIP*, 1 *AP2/ERF-ERF*, 7 zinc fingers, 2 *GARP-ARR-B*, and 1 *GARS* (**Supplementary Figure S6B**). Blasted against the TAIR database, *PB.15903.1*, *PB.22598.1*, and *PB.30746.1*, belonging to *bHLH* transcription factor, may involve in light regulated flavonoid biosynthesis. *PB.4531.3* belonging to the *DBB* transcription factor, is related to the negative regulation of light signal transduction. *PB.24525.1*, *PB.4604.1* and *PB.13554.1* related to the hormone regulation of blueberry, belong to *AUX/IAA*, zinc fingers and GRAS transcription factor, respectively. *PB.8456.1* belonging to *GARP-ARR-B* translation factor is presumably related to nitrogen synthesis of blueberry. These transcription factors may inhibit the expression of structural genes involved in flavonoid synthesis by participating in blueberry response to light, hormones, nitrogen, and other signals.

### Candidate hub genes related to flavonoid synthesis

**Figure 7:**
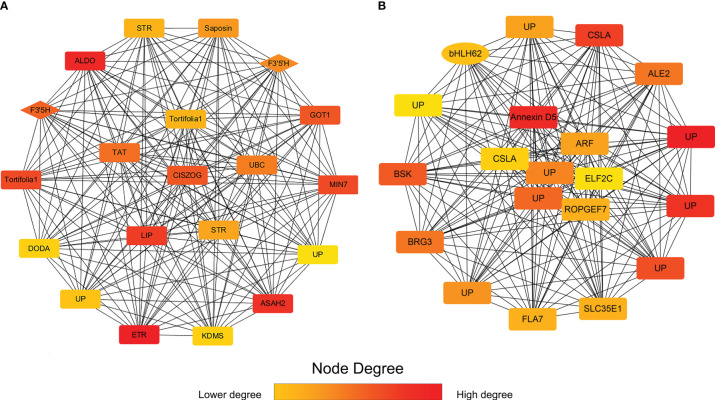
The first 20 gene networks in darkviolet and biseque4 modules. **(A)** The first 20 gene network in the darkviolet module. The diamond represents the *F3*′*5*′*H* gene. **(B)** The first 20 gene network in the bisque4 module. Ellipse represents the *bHLH* transcription factor. The more forward ranking is represented by a redder color.

To study the hub genes in darkviolet and bisque4 modules, we obtained the first 20 genes of the two modules using MCC method in the cytoHubba plugin and annotated them (**Figure 7**; **Supplementary Table S6**). Two *F3’5’H* genes (*PB.12741.1* and *PB.12745.3*) in the darkviolet module and one *bHLH* transcription factor (*PB.15903.1*) in the bisque4 module were key synthetic and regulated genes in the flavonoid synthesis pathway (**Table S7**). Blasting against TAIR database, *PB.15903.1* is the homologous sequence of *CIB2* interacting with *CRY2*, which may be related to the light regulation of flavonoid synthesis. Other genes may also be related to the synthesis and regulation of flavonoids, which needs further verification.

## Discussion

Blueberry is the most popular small blue fruit and its unique color is closely related to the type and content of flavonoids (Dong et al., 2019). Flavonoids in blueberries show excellent physiological activities, including antioxidant, anticancer, and antidiabetic activities, and reduce cardiovascular disease (Cappai et al., 2018). The high content of flavonoids, especially anthocyanins, is believed to be responsible for the health benefits of these blueberries (Rodriguez-Mateos et al., 2012). Therefore, the determination of flavonoids at different developmental stages of blueberry is not only helpful to analyze the coloring mechanism of blueberry but is also of great significance for the development of health products containing blueberry flavonoids. In this study, the UPLC–MS/MS method was used to assess the flavonoid profiles of three blueberry varieties at three typical developmental stages. The results showed that there were significant differences in the types and contents of flavonoids in different varieties and developmental stages. Among them, the highest metabolite content and the largest differential metabolites of blueberry varieties at different developmental stages were delphinidin glycosides, which is consistent with previous reports (Timmers et al., 2017; Chai et al., 2021). It is noteworthy that myricetin-3-*O*-galactoside (mvgal) has the highest flavonoid content in Duke, while delphinidin-3-*O*-galactoside has the highest flavonoid content in Misty and Emerald. According to Günther’s study, malvidin-3-*O*-glycosides were predominant in northern highbush blueberry Nui and rabbiteye Velluto Blue (Günther et al., 2020). Meanwhile, the least differential metabolites were found between Misty and Emerald among the three blueberry varieties. The flavonoid content of blueberries is affected by variety, light, temperature, environment, and other factors (Li X et al., 2019). It owes to the fact that Misty and Emerald both belong to southern highbush blueberry, and the number of differential metabolites between them was lowest compared with the remaining pairwise comparisons.

Among the new blueberry varieties announced in the past decade, the southern highbush blueberry has developed the largest number of new varieties compared with other species, which leads the main breeding direction of blueberry breeding at present. Although, the tetraploid northern highbush blueberry *V. corymbosum* has been published (Colle et al., 2019), and the transcriptome sequencing results of some blueberry varieties have been completed successively (Zifkin et al., 2012; Tang et al., 2021), which has greatly promoted the identification of candidate genes and pathways for excellent fruit quality and accelerated the molecular breeding of northern *Vaccinium* species. However, compared with the northern highbush blueberry, the southern highbush blueberry has a complex genetic background, its tetraploid genome and transcriptome resources are insufficient, and there are few researches on key quality factor genes such as anthocyanin and their regulatory factors, which seriously restricts the cultivation of the southern highbush blueberry in China (Rowland et al., 2012; Ge et al., 2019). To better understand the molecular mechanism of flavonoid synthesis, PacBio sequencing was used for the very first time in tetraploid southern highbush blueberry Misty at three fruit development stages, which provides a new interpretation of FL transcripts, variable shear, lncRNAs, and transcription factors of tetraploid southern highbush blueberry. We combined the Illumina data of the two other blueberry varieties from three different periods to analyze the DEGs of the three different varieties and three different periods. The results showed that the maximum number of DEGs was identified when compared with the green fruit stage in different varieties or different developmental stages, which is consistent with previous results (Lin et al., 2018). During the development of blueberry fruit from the green to blue fruit stage, significant changes have taken place in sugar and organic acids (Li X et al., 2020), fruit firmness (Cappai et al., 2018), fruit size, single fruit weight (Yang et al., 2021), and flavonoids (Spinardi et al., 2019). The early development of blueberry is not only involved in the development process of blueberry but also involved in the quality change of blueberry. Therefore, the differentially expressed genes are the most abundant when compared with the green fruit stage in different varieties or different developmental stages.

The synthesis of flavonoids includes phenylalanine, flavonoid, anthocyanin, flavone, and flavonol biosynthesis pathways, involving a variety of structural and regulated genes (Yi et al., 2021). Through WGCNA of differential gene and flavonoid data, two modules darkviolet and bisque4 were obtained. Nine structural genes for flavonoid synthesis were disclosed in the two modules, of which the *F3’5’H* gene in darkviolet module was identified as the key gene for flavonoid synthesis. In the present study, the expression levels of nine structural genes in Misty were significantly higher than those in Duke. Dihydroquercetin is an important precursor and node in different types of anthocyanin biosynthesis. The *F3’5’H* gene hydroxylates the B-rings of anthocyanidins (Zhang et al., 2014; Yi et al., 2021). Therefore, the high expression of *F3’5’H* genes promotes the accumulation of delphinidin and its derivatives, which are the precursors of blue and reddish-purple pigments (Zhang et al., 2014; Yi et al., 2021). Additionally, the differential gene expression of *LDOX* and *UFGT* in southern and northern highbush blueberry varieties contributed to the enrichment of delphinidin-3-*O*-galactoside in southern highbush varieties and malvidin-3-*O*-galactoside in northern highbush blueberry varieties. The glycosylation and side-chain modification of anthocyanins not only affect the stability of anthocyanins in a natural state but are also related to the color presentation of anthocyanins (Zhang et al., 2014). In this experiment, the structural gene in the negative regulation module is *LAR*, which promotes the synthesis of proanthocyanidins. It should be noted that there is a competitive relationship between proanthocyanidin and anthocyanin synthesis since they share common precursors (Karppinen et al., 2021).

The MBW complex directly regulates the transcription of flavonoid structural genes. The *MYB* transcription factor is the core transcription factor in MBW complex. The *MYB* transcription factor can regulate specific structural genes, such as the *R2R3-MYB* transcription factor, which is responsible for these specificities through conserved N-terminal R2 and R3 DNA-binding domains. In our study, *PB. 9559.1* was a homologous gene of *MYB1*. In litchi and apple, the targeted regulatory gene of *MYB1* is *UFGT* (Lai et al., 2014). In garlic shoot tissue, the co-bombardment with *MYB1* and *bHLH* (*Zm-Lc*) constructs allowed earlier development of anthocyanin pigmentation (Schwinn et al., 2016). Moreover, some transcription factors are participated in signaling pathways from the environment and hormone perception to flavonoid activation or inhibition. As the most important environmental signal, light plays an important role in the synthesis of flavonoids. In this study, we found several transcription factors might involve in this pathway. The hub gene *PB.15903.1* was found to involve in the photoreceptor *CRY2* signaling pathway. CRYs detecting blue light are related to the expression levels of *F3H*, *F3′5 ′H*, *CHS*, and *CHI* (Zheng et al., 2019). Blasted against the TAIR database, *PB.22598.1* and *PB.30746.1* act as negative regulators interacting with photoreceptors *phyA* and *phyB*. It is reported that PHY absorbs red light and far-red light, and *CHS* is not expressed in the *PHY* mutant of *Arabidopsis* (Li et al., 2018). *PB.5700.2* encodings a homolog of *HY5* (*HYH*) may participate in *phyB* signaling pathway. *HY5* has been reported to antagonize the light-regulated central inhibitor *COP1* or directly regulate the expression of *CHS*, *CHI*, *F3H*, *F3’H*, *DFR*, and *LDOX* genes (Jaegle et al., 2016). *PB.4531*, belonging to the DBB family, has directly or indirectly undergone *COP1-*mediated ubiquitination and degradation (Xiong et al., 2019). The regulation of jasmonate, auxin, trigonolactone, and gibberellin in the synthesis of flavonoids is relatively clear (LaFountain and Yuan, 2021).These plant hormones all involve a Skp1/Cullin/F-box (SCF) E3 ubiquitin ligase complex, and trigger the degradation of specific hormone receptors, negatively regulating anthocyanin accumulation by interacting with the MBW complex. In this study, *PB.4604.1*, *PB.24525.1*, and *PB.19524.2* were related to jasmonate, auxin, and brassinosteroid signaling pathways, respectively. It is reported that gibberellin enhances and inhibits the biosynthesis of anthocyanins in different species and tissues through different types of DELLA protein (LaFountain and Yuan, 2021). As a gibberellin signaling repressor in *Arabidopsis*, DELLA proteins bind to MYBL2 and JAZ proteins, resulting in higher MBW complex activities (Xie et al., 2016). *PB.13554.1* and *PB.3980.1* might participate in the gibberellin signaling pathway. In addition, some transcription factors, such as *AP2/ERF* (Zhao et al., 2021), *HD-ZIP* (Wang et al., 2020), *MYB*-related (Nguyen and Lee, 2016), and *NAC* (LaFountain and Yuan, 2021), were also found to play a role in transcriptional activation or inhibition in flavonoid synthesis. These transcription factors were also identified in our study. In this sense, this study will help to elaborate the molecular mechanism of color transformation over the course of blueberry development.

## Data availability statement

The datasets presented in this study can be found the NCBI Sequence Read Archive (SRA) platform (http://www.ncbi.nlm.nih.gov/sra/) under accession numbers from SRX14097933 to SRX14097959 of Bioproject PRJNA804018.

## Author contributions

YL, GX, and JL (7^th^ author) conceived and designed the experiment. JL (7^th^ author) detected the content of flavonoids. YL, HL, JL (4^th^ author), SW, and SB analyzed the data. YL wrote the manuscript and all authors reviewed the manuscript. All authors contributed to the article and approved the submitted version.
