# Supplementary material for: Metabolomic and transcriptomic analyses of the flavonoid biosynthetic pathway in blueberry (Vaccinium spp.)
Source: Front Plant Sci. 2023 Apr 20;14:1082245. doi: 10.3389/fpls.2023.1082245 (PMC10157174; doi:10.3389/fpls.2023.1082245)
Supplement: Supplementary file 1 [file DataSheet_1.zip › Supplemental Material-20230312/Supplementary_Material-20230120.docx]

**
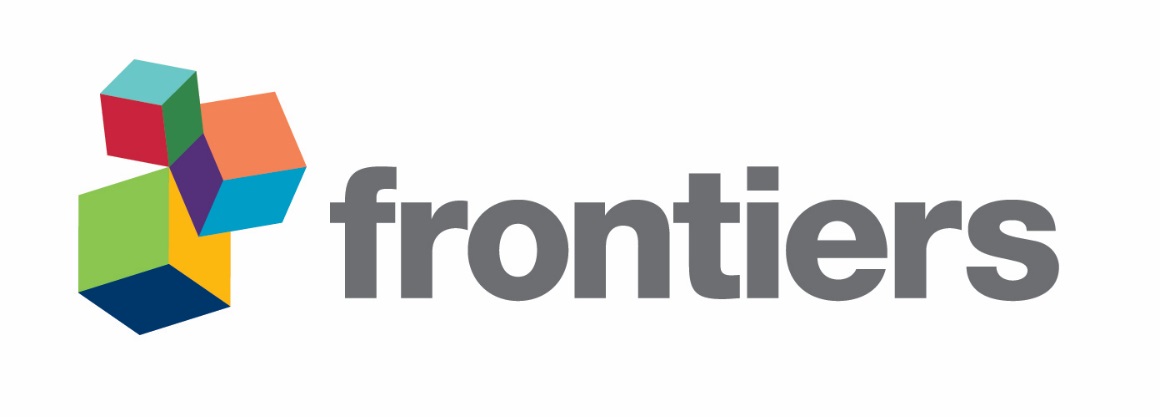
**

**Supplementary Figure 1.** Length distribution of the blueberry variety Misty obtained by SMRT sequencing on the PacBio sequel platform. **(A)** The read length distribution of ROI. **(B)** The read length distribution of FLNC. **(C)** The read length distribution of consensus isoforms.

# Supplementary Figure 2. SSRs, gene structures, IncRNAs identification, and transcription factors predicted by the full-length transcript data. (A) SSR type distribution. (B) Predicted CDS-encoded protein length distribution. (C) Venn diagram of lncRNAs predicted by CNCI, CPC, Pfam and CPAT methods. (D) Transcription factor type distribution.

**Supplementary Figure 3.** The unigenes functionally annotated using the data from seven nucleotide and protein databases (NR, GO, COG, KOG, KEGG, SwissProt and eggNOG). **(A)** Statistical table of annotated transcripts. **(B)** NR homologous species distribution. **(C)** GO function classification of the consensus sequence. **(D)** COG function classification of the consensus sequence. **(E)** KOG function classification of the consensus sequence. **(F)** eggNOG function classification of the consensus sequence.

**Supplementary Figure 4.** Comparison of the differentially expressed genes in each sample pair. DG, Duke green stage; DP, Duke pink stage; DB, Duke blue stage; EG, Emerald green stage; EP, Emerald pink stage; EB, Emerald blue stage; MB, Misty green stage; MP, Misty pink stage; MB, Misty blue stage.

**Supplementary Figure 5.** Cluster dendrogram showing 12 modules of coexpressed genes by WGCNA.

**Supplementary Figure 6.** Heatmap of all candidate genes in flavonoid biosynthesis of darkviolet and bisque4 modules. (**A**) Heatmap of all candidate genes in flavonoid biosynthesis of darkviolet module. (**B**) Heatmap of all candidate genes in flavonoid biosynthesis of bisque4 module. DG, Duke green stage; DP, Duke pink stage; DB, Duke blue stage; EG, Emerald green stage; EP, Emerald pink stage; EB, Emerald blue stage; MB, Misty green stage; MP, Misty pink stage; MB, Misty blue stage.

**Supplementary Figure 7.** Phylogenetic analysis of *VcMYBM1* and flavonoid-related *MYBs* in other species.
