# Supplementary figures and images for: Metabolomic and transcriptomic analyses of the flavonoid biosynthetic pathway in blueberry (Vaccinium spp.)
Source: Front Plant Sci. 2023 Apr 20;14:1082245. doi: 10.3389/fpls.2023.1082245 (PMC10157174; doi:10.3389/fpls.2023.1082245)

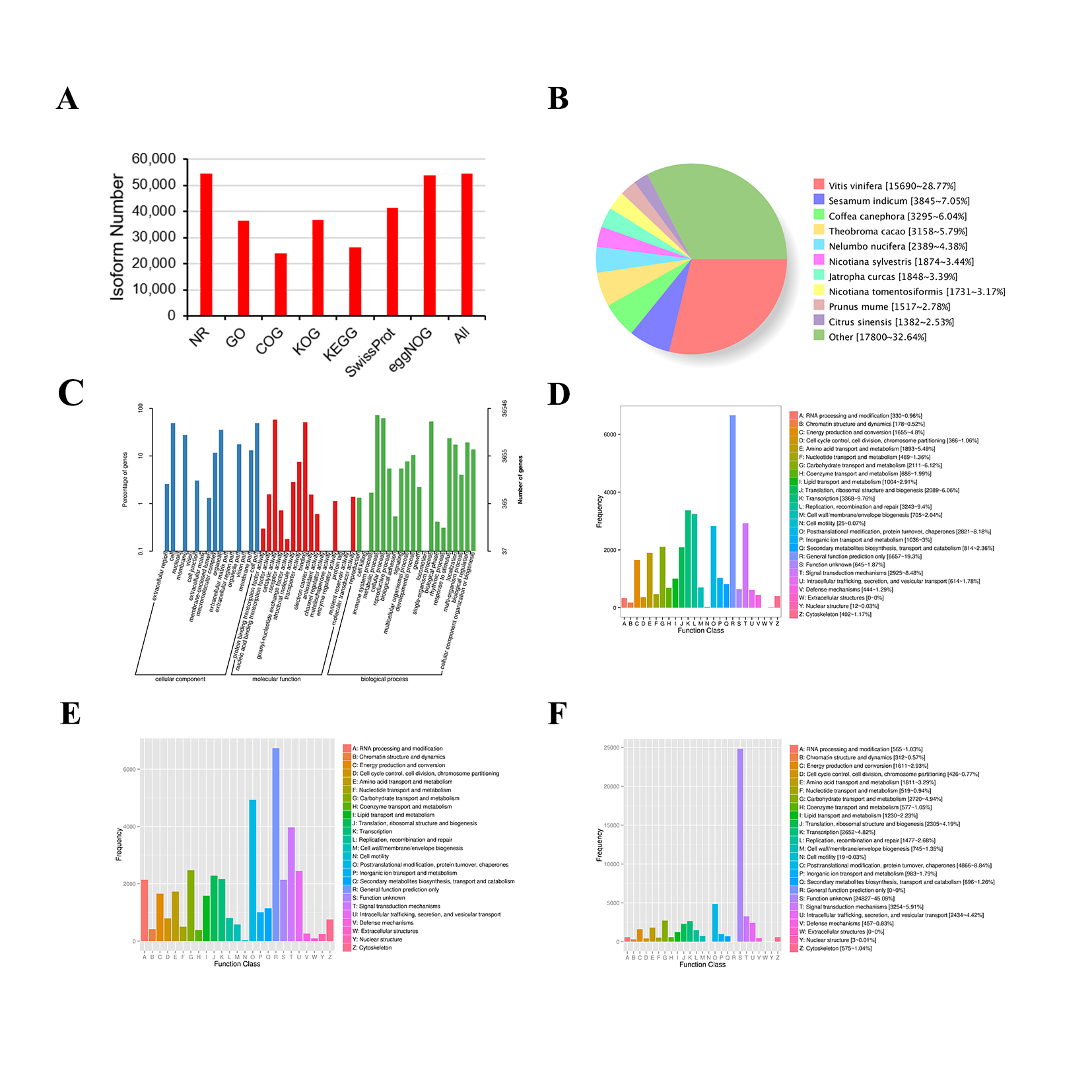

Supplement: Supplementary file 1 [file DataSheet_1.zip › Supplemental Material-20230312/FS3.tif]

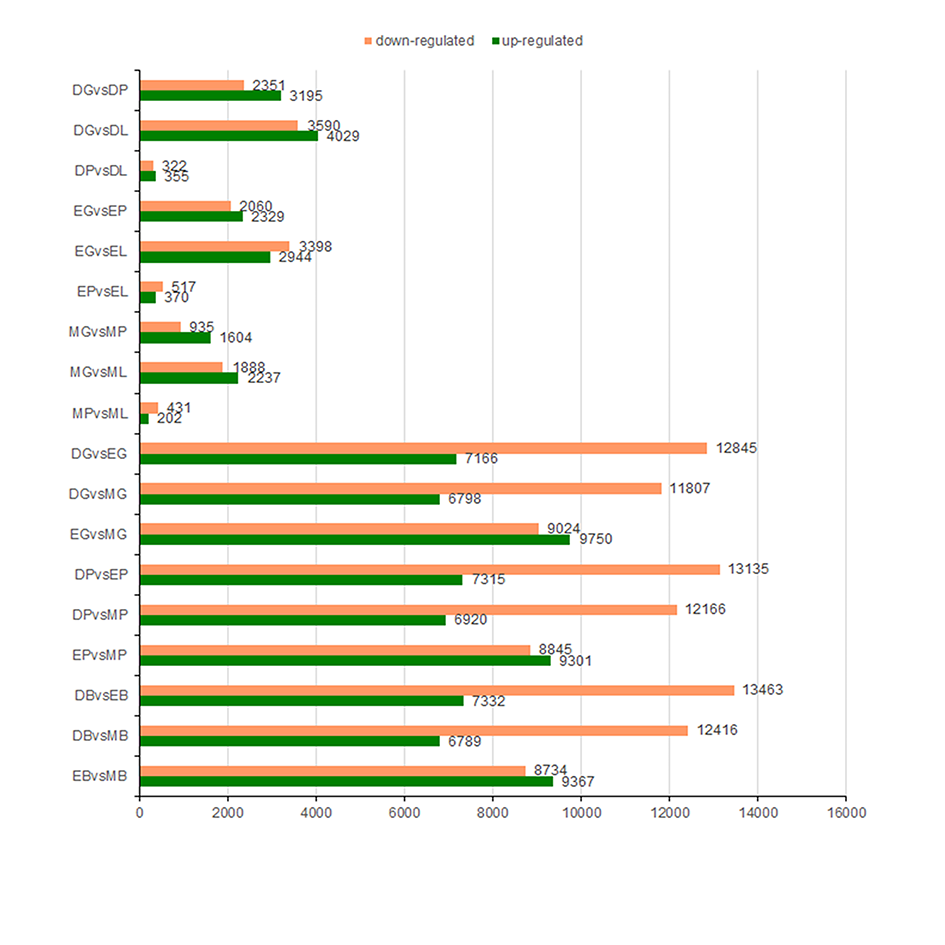

Supplement: Supplementary file 1 [file DataSheet_1.zip › Supplemental Material-20230312/FS4 .tif]

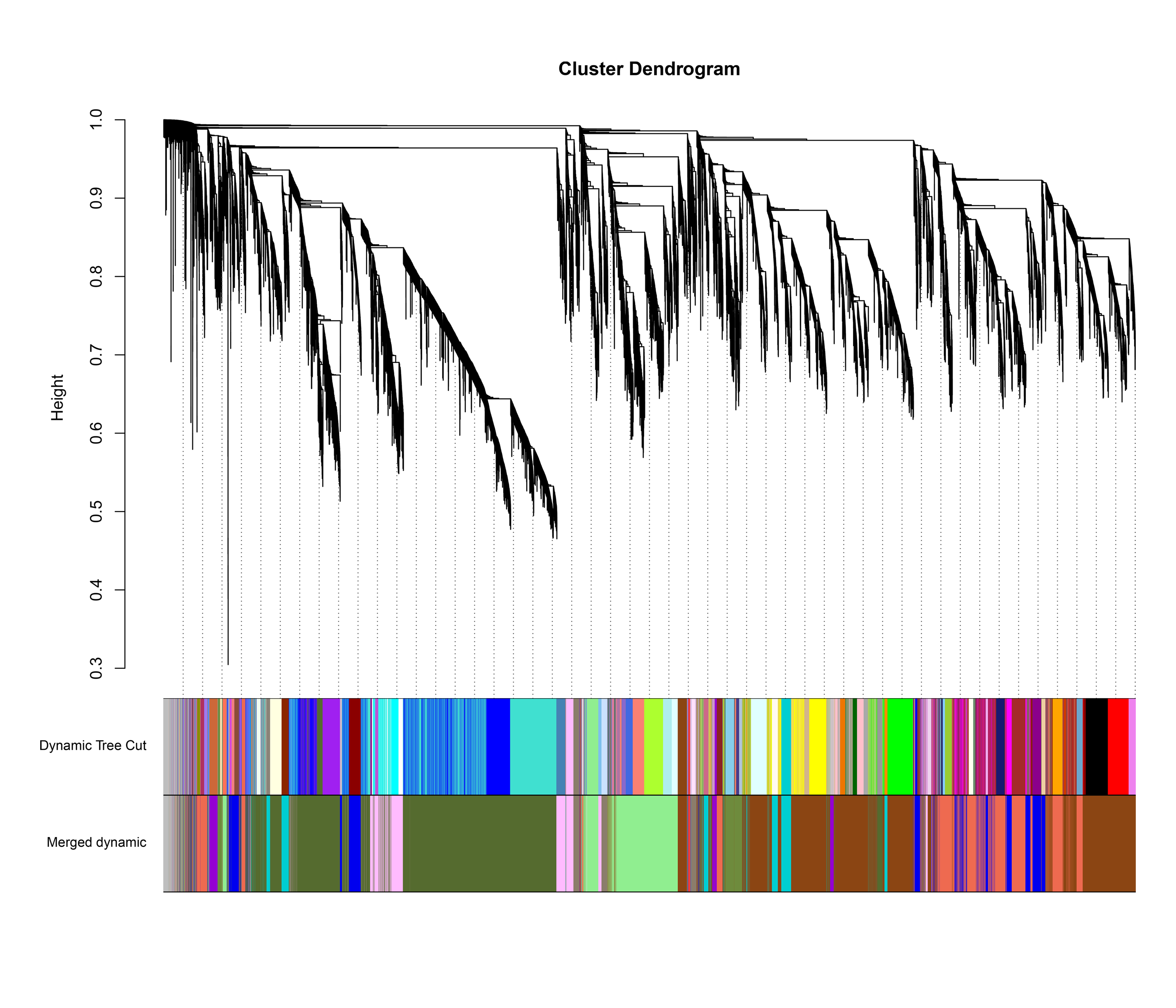

Supplement: Supplementary file 1 [file DataSheet_1.zip › Supplemental Material-20230312/FS5.tif]

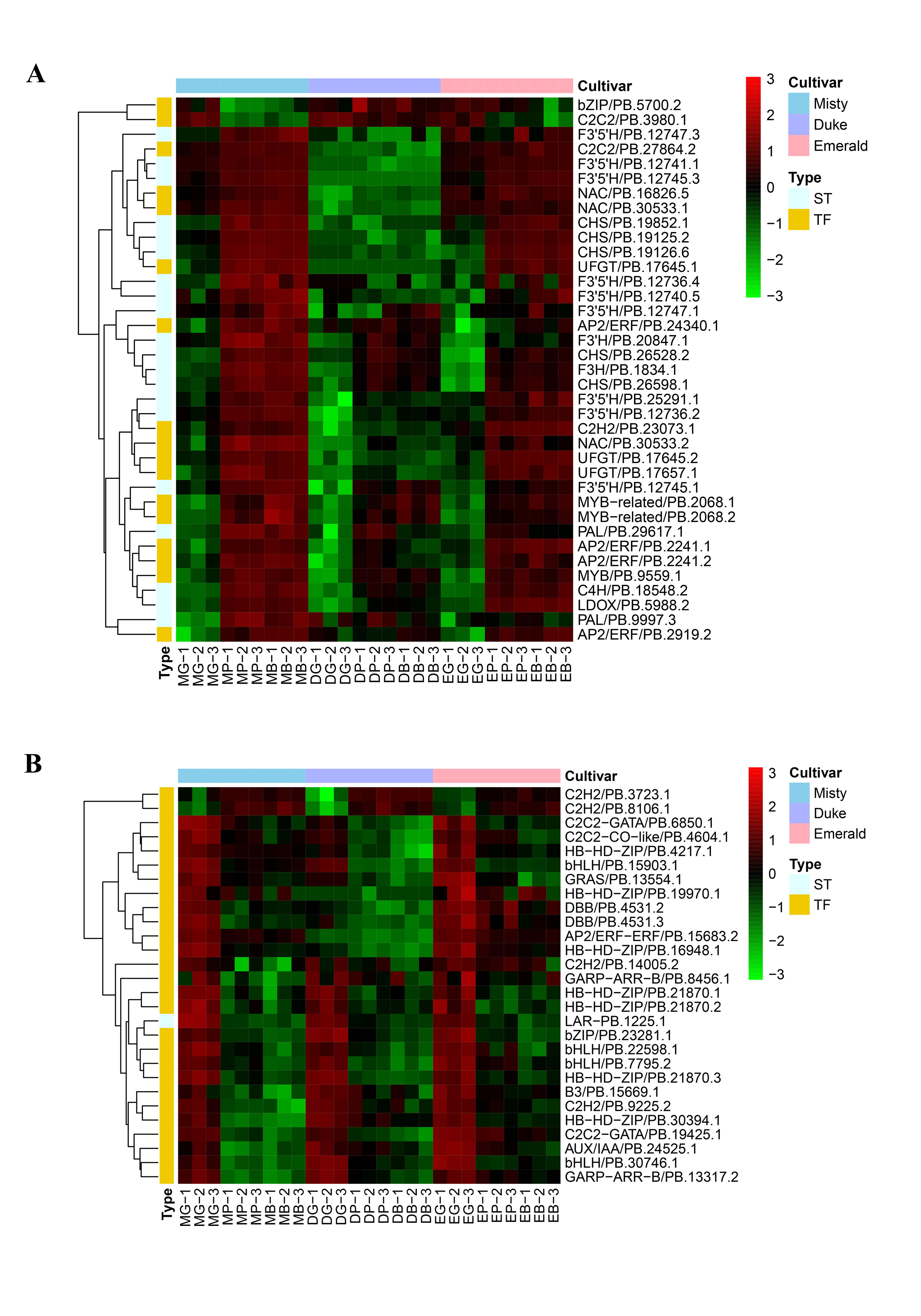

Supplement: Supplementary file 1 [file DataSheet_1.zip › Supplemental Material-20230312/FS6.tif]

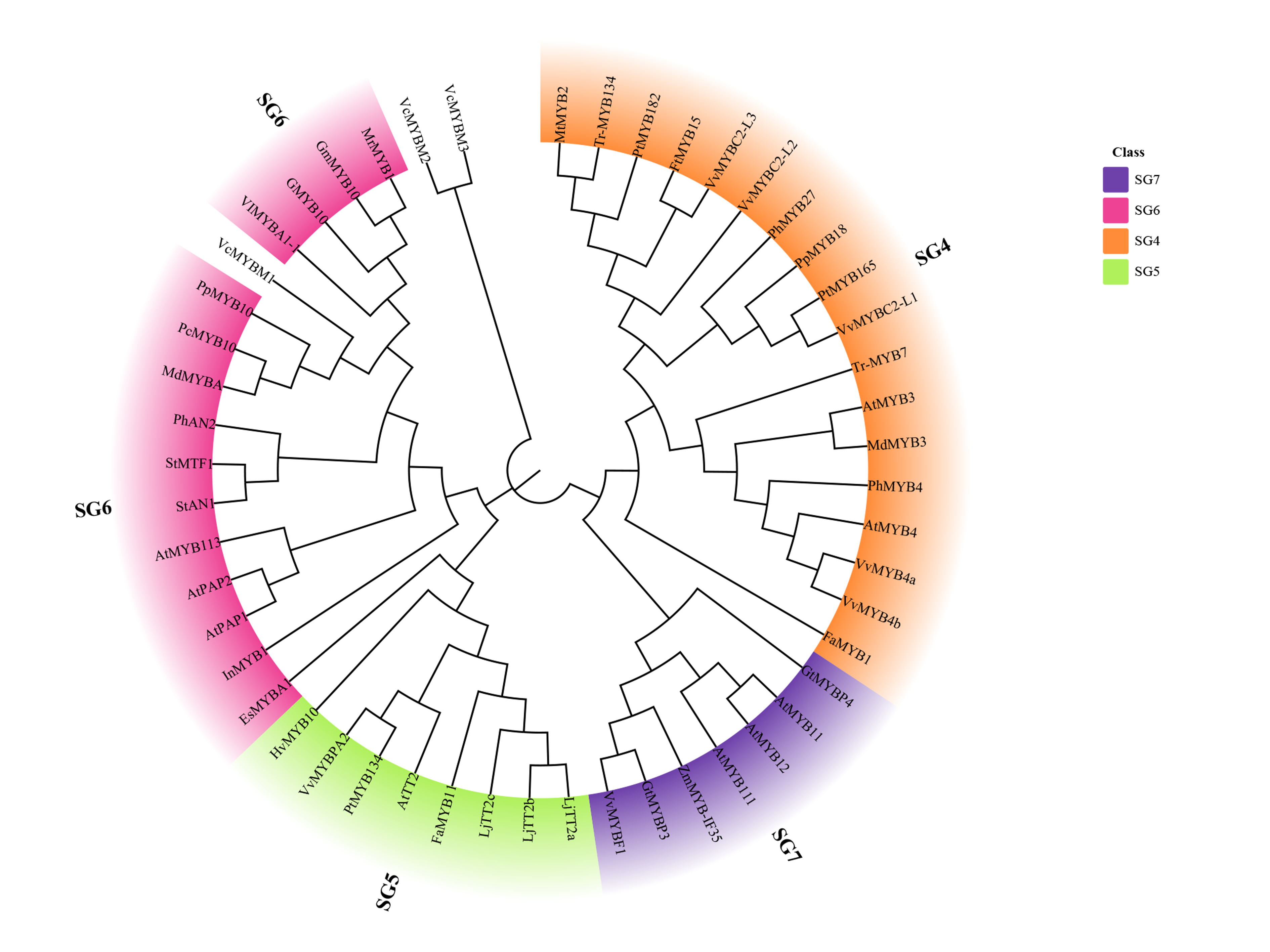

Supplement: Supplementary file 1 [file DataSheet_1.zip › Supplemental Material-20230312/FS7.tif]
